# Supplementary material for: MetaRibo-Seq measures translation in microbiomes
Source: Nat Commun. 2020 Jun 29;11:3268. doi: 10.1038/s41467-020-17081-z (PMC7324362; doi:10.1038/s41467-020-17081-z)
Supplement: Supplementary file 10 — Supplementary Data 7 [file 41467_2020_17081_MOESM10_ESM.zip › File2/Confidence_High_Taxonomy/139211_out.krona.html]

Javascript must be enabled to view this page.

members
magnitude
magnitudeUnassigned
count
unassigned
taxon
rank

139211\_out

9

2
superkingdom
9

9
phylum
976

9
class
200643

9
order
171549

815
family
9

816
9
genus

species
1
2292280

SRS148874\_contig\_number\_13848


SRS098881\_contig\_number\_3594
46506
species
1

5
species
28116

SRS019068\_contig\_number\_61310SRS019381\_contig\_number\_1391SRS053398\_contig\_number\_2193SRS058268\_contig\_number\_407SRS105153\_contig\_number\_18097


SRS048981\_contig\_number\_4463
371601
1
species

1
species

SRS024625\_contig\_number\_14474
556259
